# The MIAmaxent R package: Variable transformation and model selection for species distribution models

**DOI:** 10.1002/ece3.5654

**Published:** 2019-09-30

**Authors:** Julien Vollering, Rune Halvorsen, Sabrina Mazzoni

**Affiliations:** ^1^ Department of Environmental Sciences Western Norway University of Applied Sciences Sogndal Norway; ^2^ Department of Research and Collections Natural History Museum University of Oslo Oslo Norway

**Keywords:** lasso regularization, Maxent, maximum entropy, species distribution modeling, subset selection, variable transformation

## Abstract

The widely used “Maxent” software for modeling species distributions from presence‐only data (Phillips et al., Ecological Modelling, 190, 2006, 231) tends to produce models with high‐predictive performance but low‐ecological interpretability, and implications of Maxent's statistical approach to variable transformation, model fitting, and model selection remain underappreciated. In particular, Maxent's approach to model selection through lasso regularization has been shown to give less parsimonious distribution models—that is, models which are more complex but not necessarily predictively better—than subset selection. In this paper, we introduce the MIAmaxent R package, which provides a statistical approach to modeling species distributions similar to Maxent's, but with subset selection instead of lasso regularization. The simpler models typically produced by subset selection are ecologically more interpretable, and making distribution models more grounded in ecological theory is a fundamental motivation for using MIAmaxent. To that end, the package executes variable transformation based on expected occurrence–environment relationships and contains tools for exploring data and interrogating models in light of knowledge of the modeled system. Additionally, MIAmaxent implements two different kinds of model fitting: maximum entropy fitting for presence‐only data and logistic regression (GLM) for presence–absence data. Unlike Maxent, MIAmaxent decouples variable transformation, model fitting, and model selection, which facilitates methodological comparisons and gives the modeler greater flexibility when choosing a statistical approach to a given distribution modeling problem.

## Graphical abstract

In this paper, we introduce the MIAmaxent R package, which provides a statistical approach to modeling species distributions similar to Maxent's, but with subset selection instead of lasso regularization. The simpler models typically produced by subset selection are ecologically more interpretable, and making distribution models more grounded in ecological theory is a fundamental motivation for using MIAmaxent. Unlike Maxent, MIAmaxent decouples variable transformation, model fitting, and model selection, which facilitates methodological comparisons and gives the modeler greater flexibility when choosing a statistical approach to a given distribution modeling problem.

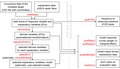

## 1 BACKGROUND

Correlative distribution models—which use spatial association to find statistical relationships between the occurrence of a modeled target and its environment—are prevalent in the ecological literature (Guisan et al., 2013). Particularly common are modeling methods that contrast presence locations against the study area at large (so‐called “background” locations), because absence data are comparatively scarce (Pearce & Boyce, 2006). One such method, maximum entropy distribution modeling, was first introduced in standalone Java software called “Maxent”, released in 2006 (Phillips, Anderson, & Schapire, 2006). The software was made freely available with a graphical user interface that is easy to use for modelers of all levels of experience. In addition, the models it produced showed high‐predictive performance in Elith et al.'s (2006) influential paper comparing various distribution modeling methods. As a result, Maxent quickly became a very popular tool for distribution modeling; the paper that introduced it has more than 5,000 citations in the Web of Science Core Collection, and more than 60% of distribution modelers report using it (Ahmed et al., 2015). Recently, the Java software (currently version 3.4.1) was also adapted into an R package called “maxnet” (Phillips, Anderson, Dudík, Schapire, & Blair, 2017, currently version 0.1.2). For the remainder of this paper, we refer primarily to the Maxent Java software, but our statements are equally applicable to the maxnet R package.

The core functionality of the Maxent software is a statistical approach that comprises three linked, main elements: (a) variable transformation (“feature creation” in Maxent's terminology), (b) maximum entropy fitting, and (c) lasso regularization. Variable transformation expands explanatory variables (EVs) into a larger set of derived variables (DVs), the maximum entropy fitting algorithm finds parameter estimates, and lasso regularization reduces model overfitting. Maxent implements these three elements together owing to its origins in the field of machine learning (Elith et al., 2011; Merow, Smith, & Silander, 2013; Phillips et al., 2006; Phillips, Dudík, & Schapire, 2004). However, there is no reason that variable transformation, maximum entropy fitting, and lasso regularization must be used in tandem; for example, a maximum entropy distribution model might equally be fit without variable transformation or by a different model selection technique. Nor is it clear that this combination of elements represents the optimal approach to presence‐background distribution modeling. In fact, the literature reveals that many models produced by Maxent are difficult to interpret and poorly grounded in ecological theory, as evidenced by highly complex response curves, large numbers of parameters, and little critical examination of modeled relationships (Halvorsen, 2013; Yackulic et al., 2013). For studies aiming to explain occurrence–environment relationships, or to project them to a different spatial or temporal context, these norms are troubling (Halvorsen, 2012, especially figure 15 therein).

The purpose of this paper is to present the MIAmaxent R package as an alternative to the Maxent software and to summarize the motivation for MIAmaxent and its underlying approach (Halvorsen, 2013; Mazzoni, Halvorsen, & Bakkestuen, 2015). First, we assess individually the utility of variable transformation, maximum entropy fitting, and lasso regularization for distribution modeling. Especially, we ask how compatible each of these elements is with ecological theory concerning species responses to environmental gradients, using insights from the long tradition of gradient analysis in ecology (Austin, 2002, 2007; Halvorsen, 2012). Then, we show how modifications to Maxent's statistical approach—implemented in MIAmaxent—lead to more ecologically grounded and interpretable distribution models. By “ecologically grounded,” we mean: in concordance with expectations about occurrence–environment relationships derived from lines of inquiry other than correlative distribution modeling. By “interpretable,” we mean: simple enough that the relationship between prediction and predictors can be explained in an ecologically meaningful way.

### 1.1 Variable transformation

Variable transformation in Maxent means that explanatory data enter into the model as DVs—functions of the original EVs supplied by the modeler (Phillips et al., 2006). It can be thought of as changing the functional form of the model specification, akin to adding polynomial terms to a linear regression. Recent versions of the software support five transformation types for continuous EVs: linear, quadratic, threshold, forward hinge, and reverse hinge (Phillips et al., 2017; Phillips & Dudík, 2008). In addition, categorical EVs are transformed into binary (dummy) DVs, and interaction terms are possible in the form of product transformations between pairs of EVs (Phillips et al., 2006). The effect of variable transformation is that the relationship between the occurrence of the modeled target and an EV can be captured more flexibly than if only the original EVs enter into the model (Austin, 2007; Halvorsen, 2013; Phillips et al., 2006). For example, if an occurrence rate is constant for one part of an EV range and monotonically increasing or decreasing for another part, then a hinge transformation of that EV allows Maxent to fit the response more closely than the original EV. Thus, variable transformation relates critically to the modeler's expectations about which model response shapes are ecologically realistic (Halvorsen, 2013; Merow et al., 2014; Phillips et al., 2006).

The gradient analysis literature shows that, across a sufficiently large interval, a species' response to any environmental determinant is generally unimodal, but truncation of the interval and the way the variable is scaled may affect the shape of the observed response (Austin, 2007; Halvorsen, 2012; Rydgren, Økland, & Økland, 2003). Thus, a particular variable transformation type accords with expectations based on gradient analysis if it enables a unimodal model response, or some truncated portion thereof, to the original EV. All of the transformation types in Maxent conform to this condition. Even a threshold transformation, which makes a continuous variable binary, can be regarded as approximating a strongly skewed and truncated unimodal response (Halvorsen, 2012). However, the list of transformation types in Maxent is not exhaustive, and other ecologically motivated transformations could also be considered (Halvorsen, 2013; Phillips et al., 2006). Increasingly, complex transformation types allow a closer fit to the data, but are less interpretable with respect to the original EV, and result in a less generalizable model.

Although the transformation types in Maxent are individually consistent with ecological expectations, combinations of the DVs they produce frequently result in model responses that are not. For example, the combined effects of multiple DVs may create a local minimum in the model response to a continuous EV (e.g., Elith et al., 2011, figure 5), which is inconsistent with the expectation of unimodality. More generally, a model containing multiple, simple transformations of a single EV can show a highly complex response to that EV. Therefore, a model will be more ecologically grounded and interpretable if the set of DVs it includes is tailored to the modeled system, based on a priori knowledge of the modeled target in the study area, or exploratory analyses of the data. Specifically, DVs that are responsible for unrealistic or unexpected response shapes, or that capture idiosyncrasies in the data rather than patterns of interest (Merow et al., 2013), should be discarded. However, Maxent applies the same transformation types to all continuous EVs and does not provide the possibility to customize the resulting set of DVs. Moreover, Maxent users frequently neglect to examine modeled relationships critically (Yackulic et al., 2013), so they are ill‐equipped to iteratively refine the set of DVs. In short, Maxent's variable transformation procedure does not offer the level of user control that is necessary to make maximally interpretable and ecologically grounded models.

### 1.2 Maximum entropy fitting

The model fitting algorithm in Maxent is based on the principle of maximum entropy (Jaynes, 1957a, 1957b), which has given the software its name. This algorithm finds the probability distribution that is minimally divergent from the distributions of predictors (equivalently: maximally uniform in space), subject to constraints given by the presence locations (Elith et al., 2011). The constraint placed on the distribution in Maxent is that the expected value of each predictor must match its empirical mean among presence locations (Phillips et al., 2006). Note that we use the term “predictor” to refer generically to any variable component of a model, regardless of whether it is transformed (DV) or not (EV). Under this constraint, the distribution which maximizes entropy follows a specific exponential distribution called a Gibbs distribution (Della Pietra, Della Pietra, & Lafferty, 1997). In recent years, it has been shown that these maximum entropy models belong to a broader family of solutions to parametric density estimation problems that also include inhomogeneous Poisson process models (IPP) and logistic regression models (Aarts, Fieberg, & Matthiopoulos, 2012; Fithian & Hastie, 2013; Renner & Warton, 2013; Warton & Shepherd, 2010). It particular, Fithian and Hastie (2013) showed that logistic regression recovers the same parameter estimates as the maximum entropy algorithm when background locations are weighted strongly compared with presence locations (so‐called “infinitely weighted logistic regression”; IWLR).

Maximum entropy fitting gives the estimate of occurrence density that is maximally noncommittal, or minimally presumptive, while conforming to the information in the data (Jaynes, 1957a). A minimally presumptive model fit may be especially appropriate for the opportunistically collected presence‐only data commonly used for distribution modeling, since frequent artifacts in these data—for example, resulting from sampling bias (Støa, Halvorsen, Mazzoni, & Gusarov, 2018)—may easily be inherited by overfitted models. For example, the classification methods (boosted regression trees and random forests) tested by Elith & Graham, (2009) were more prone than Maxent to capture idiosyncratic patterns specific to the sample, despite applying variance reduction measures similar to Maxent's lasso regularization (Elith & Graham, 2009, supplemental information). Put another way, a maximally noncommittal model is likely to generalize well beyond its training data. This fact may not be captured in measures of predictive performance, which are typically estimated with data that are not fully independent of the training data (Halvorsen, 2012) due to spatial and temporal autocorrelation in geographic distributions (Araújo, Pearson, Thuiller, & Erhard, 2005). Presence–absence evaluation data that are collected independently of the training data may nonetheless give the best indication of a model's generalizability (e.g., Edvardsen, Bakkestuen, & Halvorsen, 2011; Halvorsen et al., 2016; Pinto et al., 2016; Searcy & Shaffer, 2014; West, Kumar, Brown, Stohlgren, & Bromberg, 2016).

While some fitting algorithms assume a distinct form of the occurrence–environment relationship, such as the rectilinear environmental space dictated by the classic BIOCLIM algorithm (Guisan & Zimmermann, 2000), maximum entropy fitting is exclusively responsive to the data. That gives maximum entropy fitting a straightforward ecological interpretation: It produces responses to predictors that deviate minimally from uniformity (i.e., no response). For this reason, a model fitted by maximum entropy will be interpretable and ecologically grounded to the same degree that the predictors in the model are, which emphasizes the importance of variable transformation. Maximum entropy fitting in itself is fully compatible with ecological theory concerning species responses to environmental gradients.

### 1.3 Lasso regularization

The Maxent software uses a technique termed “lasso” (Least Absolute Shrinkage and Selection Operator) regularization to prevent model overfitting and improve model generalizability (Phillips et al., 2006; Tibshirani, 1996). Lasso regularization is one way to optimize the bias‐variance tradeoff inherent in model selection (Hastie, Tibshirani, & Friedman, 2009); a more familiar model selection technique for many ecologists is subset selection (Zuur, Ieno, & Smith, 2007). Like subset selection, lasso regularization balances a model's goodness of fit against its complexity, by penalizing model complexity. However, unlike subset selection, the penalty is on the sum of absolute values of predictor coefficients, rather than on the number of predictors (Reineking & Schröder, 2006). As a result, lasso regularization reduces the magnitude of (i.e., shrinks) coefficients, potentially to zero, instead of selecting a subset of predictors. Subset selection “is a discrete process—variables are either retained or discarded,” while “shrinkage methods [like lasso regularization] are more continuous” (Hastie et al., 2009). Lasso regularization stems from machine learning, a tradition where predictive power is valued above all else (Breiman, 2001), whereas subset selection stems from the classical tradition of hypothesis testing and aims to distinguish important predictors from insignificant ones (Hastie et al., 2009).

An important difference between lasso regularization and subset selection is that subset selection provides unbiased parameter estimates (Halvorsen, Mazzoni, Bryn, & Bakkestuen, 2015; Merow et al., 2013; Appendix A1). In contrast, lasso regularization allows the expected value of a given predictor to deviate from its mean presence value to achieve parsimony; that is, it relaxes the constraint on the maximum entropy distribution that expected values match their unbiased estimates (Halvorsen, 2013; Reineking & Schröder, 2006). This behavior is by design (Efron, 2001) and may be excused on the grounds that the constraint should not be enforced too closely, to prevent overfitting (e.g., Elith et al., 2011). However, subset selection also prevents overfitting, without biased estimates. Therefore, subset selection accords better with the principle of maximum entropy, which seeks the “least biased estimate possible on the given information” (Jaynes, 1957a), if the given information comprises the occurrence data and the predictors in the model. We emphasize that both lasso regularization and subset selection methods are subject to the bias‐variance tradeoff, so—regardless of which method is employed—model complexity needs to be optimized from case to case according to the purposes of the study and characteristics of the data (Halvorsen, 2013; Merow et al., 2014; Reineking & Schröder, 2006).

Regression simulations have shown that subset selection performs better than lasso regularization when two conditions are fulfilled: (a) Relatively few candidate predictors have an effect on the modeled target and (b) their signal in the data is sufficiently strong (Reineking & Schröder, 2006; Tibshirani, 1996). Specifically, Reineking and Schröder (2006) showed that subset selection of logistic regression models results in better discrimination ability and better identification of true predictors when less than eight of 16 candidate predictors drive the response and more than 20 presences or absences per candidate predictor are observed. Their study used presence–absence data, so its conclusions are not directly generalizable to presence‐background models, but it indicates which modeling circumstances are favorable for subset selection. Regarding species distributions, the gradient analysis literature suggests that the vast majority of environmentally determined variation in species occurrences is governed by a very limited number of composite, complex gradients (up to 3; Halvorsen, 2012). Together, these findings suggest that some distribution modeling applications may achieve better discrimination ability and better identification of true predictors via subset selection than via lasso regularization. We note that Gastón and García‐Viñas (2011) found presence‐background models selected by regularization to discriminate better than those selected by subset selection, but differences in variable transformation and interaction terms confound the comparison in that study.

One way to articulate the difference between lasso regularization and subset selection is that under lasso regularization, selection of predictors is secondary to parameter estimation, while under subset selection, parameter estimation is secondary to selection of predictors. Since predictors in distribution models usually represent ecologically meaningful quantities—especially when the purpose is to gain insight into the modeled target's response to its environment—subset selection will generally result in more interpretable models. Conceptually, if there exist two models with equal goodness of fit, where the first contains one predictor and the second contains two other predictors, and the sum of the absolute values of the coefficients in the two models are equal, then lasso regularization makes no distinction between the two models while subset selection prefers the model with only one predictor. For this reason, lasso regularization is more likely to result in models that include ecologically meaningless predictors (Halvorsen, 2013). Indeed, comparisons between lasso regularization and subset selection show that subset selection generally results in models with fewer predictors (Reineking & Schröder, 2006; Halvorsen, 2013; Halvorsen et al., 2016; Mazzoni, 2016 [chapter 6]; Appendix A2). Halvorsen et al. (2016) found that, across a range of model complexity penalties, distribution models selected by either lasso regularization or subset selection showed approximately equal predictive performance on independent presence–absence evaluation data, but the models selected by subset selection included significantly fewer predictors (see also Mazzoni, 2016, paper 6). In effect, the models selected by subset selection were more parsimonious. Lower model complexity is generally favorable for large spatial extents and coarse spatial resolutions, for small sample sizes, for strong sampling bias, and for applications that involve spatial or temporal extrapolation (Bell & Schlaepfer, 2016; Elith, Kearney, & Phillips, 2010; Merow et al., 2014; Moreno‐Amat et al., 2015; Randin et al., 2006). For example, when projecting to a new area, changes in the covariance structure of predictors (Dormann et al., 2013) pose less of a risk for models containing fewer EVs. Simpler distribution models are also more suitable for hypothesis testing than for hypothesis generation and are clearly preferable when ecological interpretation is of interest in addition to spatial prediction (Halvorsen, 2013; e.g., Bendiksby, Mazzoni, Jørgensen, Halvorsen, & Holien, 2014; Merow et al., 2014). In summary, lasso regularization's focus on prediction error promotes complexity and sacrifices ecological interpretability of distribution models.

### 1.4 Motivation for MIAmaxent

There is no consensus in the literature about the relative merits of Maxent's three core modeling elements—variable transformation, maximum entropy fitting, and lasso regularization—with regards to predictive performance or otherwise (Fletcher & Fortin, 2018; Phillips & Dudík, 2008). However, adjustments can be made to Maxent's variable transformation procedure to bring models more in line with expected ecological responses, and there is evidence that lasso regularization is not the optimal model selection technique for many distribution modeling applications (Halvorsen, 2013; Halvorsen et al., 2015, 2016; Reineking & Schröder, 2006). Applications focused on explaining the relationship of the modeled target to its environment, rather than predicting the modeled target's distribution in geographic space (Araújo et al., 2019; Halvorsen, 2012), are better served by subset selection than lasso regularization, because the former produces more interpretable models. Applications focused on projecting model predictions outside of the spatial or temporal context of the data may also benefit from subset selection, because it tends to result in lower model complexity, which often improves model generalizability (Bell & Schlaepfer, 2016; Elith et al., 2010; Merow et al., 2014; Moreno‐Amat et al., 2015).

While it is possible to use the Maxent software without lasso regularization, practically none do so (Halvorsen, 2013; Mazzoni et al., 2015; Morales, Fernández, & Baca‐González, 2017). This is unsurprising, because in the absence of an alternative, turning off lasso regularization will result in overfitted, highly complex models. Therefore, there is a need for a tool that replaces lasso regularization with subset selection, while retaining the two other core elements of Maxent. Software availability strongly affects modeling decisions (Ahmed et al., 2015), so a tool for subset selection will increase the likelihood that modelers investigate alternatives to lasso regularization. Furthermore, the coupling of variable transformation, maximum entropy fitting, and lasso regularization in the Maxent software hinders proper investigation of alternatives to each. Decoupling these three statistical elements will improve methodological comparisons and thereby advance good distribution modeling practice (Elith & Graham, 2009; Golding et al., 2017; Halvorsen et al., 2015; Mazzoni, 2016; Naimi & Araújo, 2016).

## 2 FUNCTIONALITY AND METHODS

### 2.1 Core functionality and novelty

**Figure 1:**
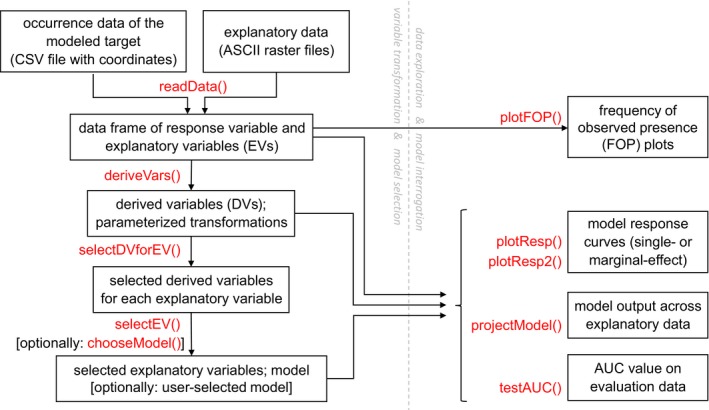
A workflow diagram for MIAmaxent, showing all top‐level functions in red, with short summaries of their inputs and outputs. Parts of the workflow involving variable transformation and model selection are on the left, while parts involving data exploration and model interrogation are on the right

The MIAmaxent R package addresses the needs described above. Its functionality primarily concerns the “statistical model” component of distribution modeling process, sensu Austin (2002, see also Halvorsen, 2012). It implements variable transformation, maximum entropy fitting, and subset selection, in a modular, adaptable manner (Mazzoni, 2016). The name “MIAmaxent” is derived from an early precursor to the package called the “MIA Toolbox” (Mazzoni et al., 2015) and signifies a “Modular Integrated Approach” to Maxent. Since maximum entropy fitting by infinitely weighted logistic regression (IWLR) is a trivial task in R, MIAmaxent's most important innovations are its implementations of variable transformation and subset selection. Additionally, MIAmaxent provides the option of using standard logistic regression in place of maximum entropy fitting by IWLR, without affecting variable transformation or subset selection. MIAmaxent's top‐level functions correspond to a workflow that runs from the training data supplied for modeling to prediction and evaluation tools (Figure 1). We note that important distribution modeling considerations independent of the statistical model—such as collection of explanatory data, conceptualization of the study area, and treatment of spatial autocorrelation or sampling bias in the occurrence data—are not addressed in MIAmaxent and must be handled separately. Sampling bias (and detection bias) deserves especially careful consideration, because it may severely handicap presence‐background models (Merow et al., 2013; Phillips et al., 2009). Model‐based sampling bias correction (e.g., Merow, Allen, Aiello‐Lammens, & Silander, 2016) is not currently implemented in MIAmaxent, so we recommend correcting sampling bias in training data prior to starting MIAmaxent's workflow, for example, by thinning presences (Aiello‐Lammens, Boria, Radosavljevic, Vilela, & Anderson, 2015).

#### 2.1.1 Variable transformation

MIAmaxent provides a standard set of transformation types that balances model flexibility and ecological interpretability. The set of transformation types implemented in the Maxent software is expanded slightly in MIAmaxent, as proposed by Halvorsen et al. (Halvorsen, 2013; Halvorsen et al., 2015; Mazzoni et al., 2015). Linear transformations are supplemented with an additional monotonous transformation—the zero‐skewness transformation (Økland, Økland, & Rydgren, 2001; Økland, Rydgren, & Økland, 2003)—to reflect the fact that the scaling of the explanatory variable (EV) may not be ecologically meaningful for the species in question (Halvorsen, 2013). Maxent's quadratic transformation is replaced with a deviation transformation, which produces scaled distances from the EV value with the highest frequency of occurrence. As a result, the unimodal response expected based on gradient analysis may be captured by a single deviation‐type derived variable (DV; Halvorsen et al., 2015). In Maxent, a unimodal response can be captured only through a combination of transformations (Halvorsen, 2013; Phillips et al., 2006). MIAmaxent's product transformations are produced during subset selection and are discussed in the following section. Like Maxent, MIAmaxent linearly rescales all DVs to the interval [0,1], which makes the magnitudes of coefficients directly comparable among variables.

In Maxent, the types of transformations applied depend on the number of presences; by default, smaller data sets are restricted to a reduced set of transformation types to avoid overfitting (Phillips & Dudík, 2008). Because the likelihood ratio tests used for subset selection in MIAmaxent account for sample size, MIAmaxent applies all transformation types by default, regardless of the size of the occurrence data set. Simultaneously, MIAmaxent offers fine control of which DVs enter into the model, as called for by Halvorsen (2013). For example, a single DV may be removed from the pool of candidates for selection if it is found to produce an ecologically unrealistic or unexpected model response. Likewise, different types of transformations may be used for different EVs, and custom DVs may be added. The Maxent software does not offer the same degree of control over variable transformation because it creates DVs and enters these into the model in a single step. Greater control of model specification especially improves MIAmaxent's utility for hypothesis testing with distribution models (Merow et al., 2014).

In addition to transforming the values of the EVs supplied by the modeler, MIAmaxent also returns the parameterized transformations as functions in R. Together with the model parameters, the parameterized transformations link EVs to model predictions and comprise a self‐contained ecological model whose predictions are easy to reproduce. Predictions from models built using the Maxent Java software (although not the maxnet R package) are much more difficult to reproduce because the parameterized transformations are not stored. An existing Maxent model can only be projected to new values of EVs by reparameterizing the model with the original training data and settings, or by reproducing its transformations based on the metadata returned by Maxent.

Finally, we emphasize that variable transformations in MIAmaxent may be used identically for models fitted by maximum entropy or logistic regression. With reference to Maxent's variable transformations, Merow et al. (2014) write: “in principle, this same complexity could be fit in a traditional GLM [logistic regression] but this is typically impractical.” MIAmaxent removes this obstacle and makes it easy to create many DVs as candidates for selection in a logistic regression model (Mazzoni et al., 2015).

#### 2.1.2 Subset selection

MIAmaxent implements subset selection in the form of forward stepwise selection. This means that model selection proceeds by adding variables to a minimal starting model, usually one by one, for as long as the improvement in goodness of fit outweighs the penalty on complexity. A significance test, which explicitly accounts for sample size, is used to compare the more complex model to its nested, simpler alternative (Halvorsen, 2013). Maximum entropy fitting of presence‐background models is a special case of classical regression methods (Fithian & Hastie, 2013), and its maximum‐likelihood interpretation may be used to derive likelihood ratio tests between nested models (Halvorsen, 2013; Halvorsen et al., 2015, appendix 2). The significance threshold (alpha) used for likelihood ratio tests may be decided a priori, or by choosing a value that maximizes some measure of fit by cross‐validation or out‐of‐sample validation. The latter has been recommended for setting the strength of lasso regularization in Maxent (Merow et al., 2013).

Forward stepwise selection is a less stable process than some other subset selection procedures, because it is a so‐called “greedy algorithm,” which selects variables in order of greatest explanatory power (Araújo & Guisan, 2006; Elith et al., 2011). Thus, selection order may affect the final result, and the algorithm will not necessarily find the universally optimal subset. Bidirectional selection is more likely to reach the universally optimal subset, and best‐subset selection will do so by definition, but these algorithms require more computation. For best‐subset selection in particular, the amount of computation quickly becomes intractable as the number of candidate predictors increases. Moreover, empirical comparisons show that forward stepwise selection very frequently yields the same or nearly the same models as bidirectional (Wiegand, 2010) or best‐subset selection (Murtaugh, 2009). Therefore, forward stepwise selection is a computationally efficient choice among forms of subset selection.

MIAmaxent is designed to carry out forward stepwise selection in a two‐stage, hierarchical procedure, executed with two different functions (selectDVforEV() and selectEV()). Because a single EV is generally transformed into multiple DVs, candidates for selection are inherently grouped according to their origin. Therefore, MIAmaxent allows the modeler to first select a parsimonious set of DVs for each EV and then select a parsimonious set of these DV sets—each representing a single EV (Halvorsen, 2013). During the second stage of the procedure, each set of DVs is treated as a unit that cannot be disaggregated, and forward selection proceeds as though the set constitutes an individual variable, while still accounting for each DV as one degree of freedom. Hastie et al. (2009) recommend this way of handling inherently grouped variables in subset selection. To be clear, EVs do not enter the model in this procedure; instead, MIAmaxent treats the selected set of DVs derived from a given EV as an integral unit that takes the place of the EV. Therefore, with regard to MIAmaxent, “selection of EVs” is to be understood as shorthand for “selection of sets of DVs representing EVs”. The hierarchical selection procedure can also be used to manipulate model complexity; for example, strict DV selection but lax EV selection will result in model with simple responses to individual EVs, but potentially many EVs.

A number of additional features in MIAmaxent improve control over the forward stepwise selection process, or aid interpretation of its outcome. First, it is possible to start EV selection from a minimal model that includes one or more EVs, by specifying a model formula. A priori inclusion of EVs may be desirable when these are known to affect the distribution (Gelman & Hill, 2006, first general principle for building regression models for prediction). It is also useful for testing hypotheses. Second, first‐order interactions between pairs of EVs with individually significant main effects may be tested. Interactions are not considered by default because their ecological justification is usually more tenuous than that of main effects (Merow et al., 2014). Third, MIAmaxent calculates and reports the fraction of null deviance explained for each model along the trail of selection (Mazzoni et al., 2015). This value is analogous to the *R* ^2^ of least squares regression and termed “*D* ^2^” in MIAmaxent, following Guisan and Zimmermann (2000). It can be used to compare nested models and to assess the relative contributions of variables in a model (Halvorsen, 2013). Note, however, that as a measure of variable contribution, these values are dependent on the order of inclusion in the model, so they should be interpreted with caution (Halvorsen et al., 2015).

Some authors in the distribution modeling literature recommend prescreening EVs to reduce collinearity between candidates for selection (e.g., Merow et al., 2013). When using the Maxent software, reduced collinearity leads to more interpretable models, since two highly correlated variables may otherwise both be retained under lasso regularization (Merow et al., 2013). With MIAmaxent, subset selection will not retain both of two highly correlated variables unless the second accounts for a significant amount of variation beyond (i.e., orthogonal to) that accounted for by the first. Therefore, prescreening does not make MIAmaxent models more interpretable, and we recommend letting likelihood ratio tests determine which of two highly correlated EVs is the better predictor, except if model predictions are made for new data with changed covariance structure. In that case, using ecological knowledge to prescreen for the more proximal variables among correlated sets may reduce the risk associated with collinearity (Austin, 2002; Dormann et al., 2013).

### 2.2 Additional functionality

#### 2.2.1 Data exploration

An intuitive way to explore environment–occurrence relationships manifested in a distribution modeling dataset is to plot occurrence rates against intervals or levels of EVs (Halvorsen, 2013). If the occurrence data comprise presence locations only, this rate reflects frequency of *observed* presence (FOP), while presence–absence data allow quantification of empirical frequency of presence (Støa et al., 2018). Hereafter, we refer only to plots of FOP, but our statements apply to both rates. Examining FOP plots is a useful data exploration step because it allows the modeler to compare prior expectations about occurrence–environment relationships to patterns in the data (Yackulic et al., 2013). FOP plots reveal patterns of occurrence specific to the study area, which may be contrary to expectations based on ecological knowledge. For example, a species generally considered thermophilic may show higher FOP at cold temperatures if the study area contains only the upper limit of its temperature range. Thus, a FOP plot may help the modeler anticipate the model's behavior. Exploring FOP patterns may also guide the choice of transformation types (Halvorsen, 2013; Merow et al., 2013). For example, threshold transformations may be turned off if FOP plots show no abrupt shifts. Furthermore, MIAmaxent's FOP plots show the relative frequency of EVs values in the dataset (i.e., data density). Modeled relationships relate critically to the distributions of EVs in the dataset (Elith & Graham, 2009), and regions of data sparsity identified in a FOP plot may be associated with increased model uncertainty.

#### 2.2.2 Model interrogation

A basic but often overlooked way to understand a distribution model is to examine its parameter estimates (Yackulic et al., 2013). Parameter estimates are easily extracted from the model object in MIAmaxent and should always be reported. Since derived variables are always scaled to [0,1], the magnitudes of their coefficients are directly comparable.

Similarly, a response curve is an important and intuitive means to evaluate the ecological plausibility of a model, and response curves should be examined even when the modeling purpose is purely spatial prediction (Guevara, Gerstner, Kass, & Anderson, 2018; Jarnevich, Stohlgren, Kumar, Morisette, & Holcombe, 2015; Merow et al., 2014, 2013). We recommend specifically that local minima in response curves be treated with skepticism, since these are often artifacts (Elith & Graham, 2009), and ecologically unlikely (Austin, 2002; Halvorsen, 2013). It is especially important to evaluate the trends of a response curve with respect to potential extrapolation; inspecting response curves can help the modeler decide whether “clamping” of predictions—whereby EV values are constrained to the interval present in the training data—is desirable (Elith et al., 2010; Guevara et al., 2018; Owens et al., 2013).

Model predictions for any supplied values of EVs are returned in the same nonspatial (data frame class) or spatial (raster class) format as the EVs. In addition, model predictions are always accompanied by the ranges of the supplied EV values compared with the training data range [0,1], which helps evaluate the risk of speculative extrapolation. Predictions from models fitted by maximum entropy are scaled to probability ratio output (PRO; Halvorsen, 2013), which can be interpreted as the “relative suitability of one place versus another” (Elith et al., 2011) and has the range (0,∞). PRO avoids the problematic assumptions inherent to Maxent's “logistic” and “cloglog” outputs (Hastie & Fithian, 2013; Merow et al., 2013; Phillips et al., 2017; Yackulic et al., 2013), as well as the scale‐dependence of Maxent's “raw” output (Halvorsen, 2013; Merow et al., 2013; Phillips & Dudík, 2008). In particular, PRO = 1 is a useful reference value that represents the relative suitability of a location randomly chosen from the entire set of training data locations used to parameterize the model, that is, the suitability of an “average” training data location (Halvorsen, 2013).

Finally, MIAmaxent can quantify a model's discrimination ability as the area under the curve (AUC) of the receiver operating characteristic (Fielding & Bell, 1997)—preferably using independent presence–absence occurrence data. Evaluation data collected independently of the training data are extremely informative and should be prioritized more often, especially in the context of projective modeling (Araújo et al., 2019; Araújo & Guisan, 2006; Bahn & McGill, 2013; Edvardsen et al., 2011; Halvorsen, 2012). Strict spatial independence between training data and evaluation data may not be achievable, since presences only occur within the spatially autocorrelated distribution of the species, but independently sampled data are a worthwhile alternative. To underline the importance of distinguishing between AUC calculated using presence‐only or presence–absence data (Yackulic et al., 2013), MIAmaxent produces a warning when calculating AUC with presence‐only data.

## 3 EXAMPLES

Both examples below are easily reproducible in R (version 3.5.2), using the R markdown file used to create this paper, which is available on GitHub (https://github.com/julienvollering/MIAmaxent-paper).

### 3.1 MIAmaxent workflow

In this section, we briefly demonstrate a basic modeling workflow in MIAmaxent (Figure 1). An expanded version of this demonstration accompanies the package as a vignette and can be accessed at: https://cran.r-project.org/web/packages/MIAmaxent/vignettes/a-modeling-example.html.

The basic data format from which all analysis in MIAmaxent proceeds is a data frame with the response variable (presence/uninformed background or presence/absence) and explanatory variables (EVs). The readData() function provides convenient data import from spatial data formats commonly used in the Maxent software (CSV coordinates and ASCII raster files using the same coordinate reference system), but may be bypassed if data are already in tabular format. The data frame we use in this example, called “traindata,” consists of 1,059 presence locations of seminatural grasslands and 16,420 uninformed background locations, together with the associated values of 13 EVs representing topography, geology, and human infrastructure.

**Figure 2:**
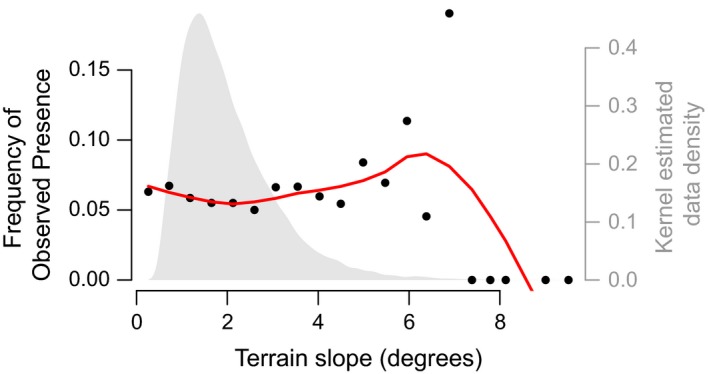
An example of a frequency of observed presence (FOP) plot for a continuous explanatory variable (EV)—in this case seminatural grassland as a function of terrain slope. The plot shows binned occurrence frequencies (black), a local polynomial regression fit of binned occurrence frequencies (red), and the density of EV values in the data set (gray)

The plotFOP() function plots frequency of observed presence (FOP) and data density across the range of a given EV. The following command produces a FOP plot (Figure 2) for terrain slope—one of the continuous EVs:

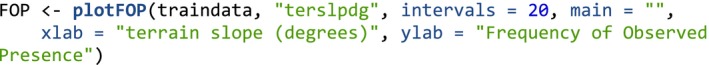

FOP plots may help guide choice of variable transformation types. Based on the plot shown here, for example, the modeler may decide to retain threshold transformations to capture the abrupt decline in observed occurrence at the highest values of the EV.

The deriveVars() function produces derived variables (DVs) from EVs by seven different transformation types: linear (L), monotonous (M), deviation (D), forward hinge (HF), reverse hinge (HR), threshold (T), and binary (B). The first six of these may be applied to continuous variables, while the binary transformation is only relevant for categorical variables. The following command applies all available transformation types (the default):

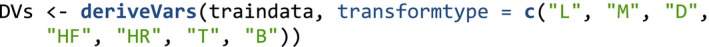

The results of deriveVars() comprise varying numbers of DVs for each EV, depending on preselection of threshold and hinge transformations of continuous variables, and on the number of levels in categorical variables. DVs for the EV shown in the FOP plot (Figure 2) consist of:

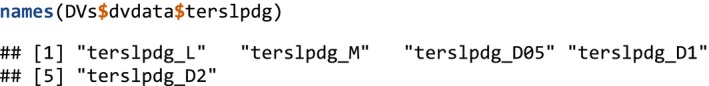

Note that, the names of DVs are embedded with metadata to indicate the type of transformation was used to create them (Mazzoni et al., 2015). For example, “terslpdg_D2” is the squared deviation from an estimated optimum in “terslpdg” (around 6). deriveVars() also returns the parameterized transformation function that was used to produce each DV.

In the first stage of model selection, the selectDVforEV() function performs forward stepwise selection separately on each group of DVs stemming from a single EV—selecting those DVs which explain a significant amount of variation in the response variable under the specified significance threshold (default alpha = 0.01):

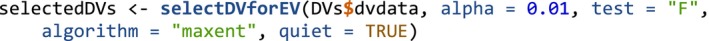

selectDVforEV() returns two list items: The DVs selected for each EV and the corresponding trail of forward stepwise selection for each EV. The trail of selection for the EV shown in the FOP plot (Figure 2) is:

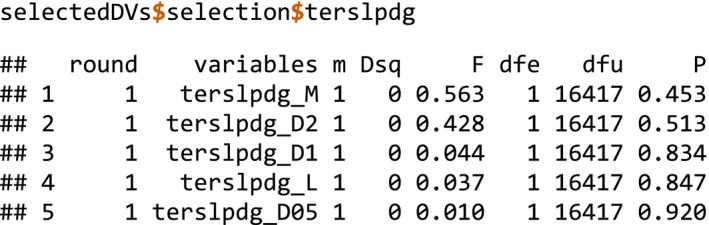

Because none of these DVs from this EV accounted for a significant amount of variation in the response variable, the EV was dropped:

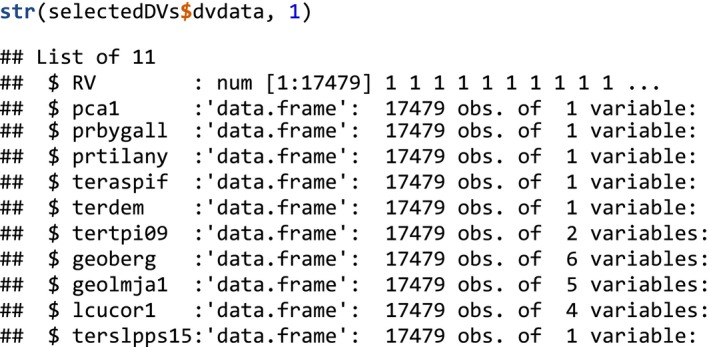

In the second stage of model selection, the selectEV() function selects whole sets of DVs stemming from a single EV—picking those sets which explain a significant amount of variation in the response variable under the specified significance threshold (default alpha = 0.01):

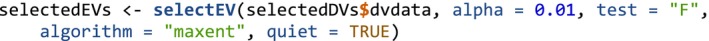

**Figure 3:**
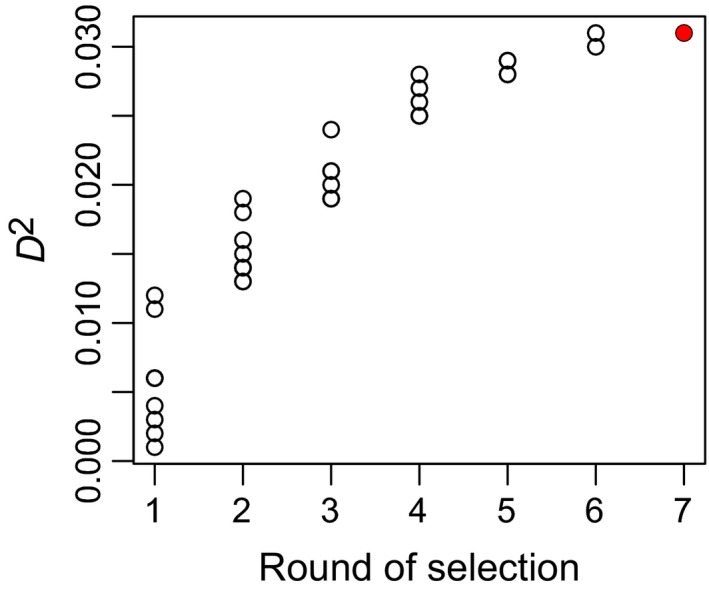
An example of the increase in fraction of null deviance accounted for (*D* ^2^) across rounds of forward stepwise selection of explanatory variables (EVs). Each round contains models obtained by adding a single EV to the best model of the previous round. The automatically selected model is shown in red

selectEV() returns three list items: the selected EVs as represented by their DVs, the trail of forward stepwise selection of EVs (Table 1), and the model automatically selected under the specified significance threshold. In this case, the automatically selected model contains 20 DVs representing 7 different EVs, and accounts for 3.1% of null deviance. Comparing this model to other models along the same trail of forward selection, the modeler may decide to proceed with the best model containing only 6 EVs, since it accounts for nearly the same fraction of null deviance (Figure 3).

**Table 1:** An example of a trail of forward selection of explanatory variables (EVs)—in this case under a significance threshold of alpha = 0.01

Round	Variables	*m*	*D*sq	*F*	*df*_e_	*df*_u_	*p*
1	prbygall	1	0.012	205.297	1	16,417	0.00e + 00
1	terslpps15	1	0.006	105.523	1	16,417	0.00e + 00
1	terdem	1	0.006	98.929	1	16,417	0.00e + 00
1	tertpi09	2	0.006	53.509	2	16,416	0.00e + 00
1	lcucor1	4	0.011	44.290	4	16,414	0.00e + 00
1	geoberg	6	0.006	17.867	6	16,412	0.00e + 00
1	pca1	1	0.003	48.486	1	16,417	0.00e + 00
1	geolmja1	5	0.004	12.075	5	16,413	0.00e + 00
1	prtilany	1	0.002	33.389	1	16,417	0.00e + 00
1	teraspif	1	0.001	14.018	1	16,417	1.82e−04
2	prbygall + lcucor1	5	0.018	24.222	4	16,413	0.00e + 00
2	prbygall + geoberg	7	0.019	18.460	6	16,411	0.00e + 00
2	prbygall + tertpi09	3	0.016	29.039	2	16,415	0.00e + 00
2	prbygall + terslpps15	2	0.015	49.130	1	16,416	0.00e + 00
2	prbygall + terdem	2	0.014	28.211	1	16,416	1.00e−07
2	prbygall + geolmja1	6	0.014	6.840	5	16,412	2.20e−06
2	prbygall + pca1	2	0.014	21.448	1	16,416	3.70e−06
2	prbygall + teraspif	2	0.013	11.441	1	16,416	7.20e−04
2	prbygall + prtilany	2	0.013	6.414	1	16,416	1.13e−02
3	prbygall + lcucor1 + geoberg	11	0.024	17.478	6	16,407	0.00e + 00
3	prbygall + lcucor1 + tertpi09	7	0.021	25.711	2	16,411	0.00e + 00
3	prbygall + lcucor1 + terslpps15	6	0.021	46.571	1	16,412	0.00e + 00
3	prbygall + lcucor1 + geolmja1	10	0.020	7.135	5	16,408	1.10e−06
3	prbygall + lcucor1 + pca1	6	0.019	12.490	1	16,412	4.10e−04
3	prbygall + lcucor1 + teraspif	6	0.019	11.395	1	16,412	7.38e−04
3	prbygall + lcucor1 + terdem	6	0.019	8.453	1	16,412	3.65e−03
4	prbygall + lcucor1 + geoberg + tertpi09	13	0.028	26.624	2	16,405	0.00e + 00
4	prbygall + lcucor1 + geoberg + terslpps15	12	0.027	48.538	1	16,406	0.00e + 00
4	prbygall + lcucor1 + geoberg + geolmja1	16	0.026	6.115	5	16,402	1.15e−05
4	prbygall + lcucor1 + geoberg + teraspif	12	0.025	13.564	1	16,406	2.31e−04
4	prbygall + lcucor1 + geoberg + pca1	12	0.025	13.063	1	16,406	3.02e−04
4	prbygall + lcucor1 + geoberg + terdem	12	0.025	4.899	1	16,406	2.69e−02
5	prbygall + lcucor1 + geoberg + tertpi09 + pca1	14	0.029	24.217	1	16,404	9.00e−07
5	prbygall + lcucor1 + geoberg + tertpi09 + geolmja1	18	0.029	5.862	5	16,400	2.04e−05
5	prbygall + lcucor1 + geoberg + tertpi09 + teraspif	14	0.028	15.906	1	16,404	6.69e−05
5	prbygall + lcucor1 + geoberg + tertpi09 + terslpps15	14	0.028	6.538	1	16,404	1.06e−02
6	prbygall + lcucor1 + geoberg + tertpi09 + pca1 + geolmja1	19	0.031	5.853	5	16,399	2.08e−05
6	prbygall + lcucor1 + geoberg + tertpi09 + pca1 + teraspif	15	0.030	13.135	1	16,403	2.91e−04
7	prbygall + lcucor1 + geoberg + tertpi09 + pca1 + geolmja1 + teraspif	20	0.031	12.353	1	16,398	4.41e−04

Note

Columns represent: the round of EV selection (“round”), the names of the EVs included in the model (“variables”), the number of DVs in the model (“*m*”), the fraction of deviance explained (“*D*sq”, sensu Guisan & Zimmermann, 2000), the *F*‐statistic for the nested model comparison (“*F*”), the degrees of freedom associated with explained deviance (“*df*_e_”) and unexplained deviance (“*df*_u_”), and the *p*‐value for the *F*‐statistic under the specified degrees of freedom (“*p*”).

The chooseModel() function returns the model specified by a supplied formula object and may be used to pick a simpler model from the trail of forward EV selection:

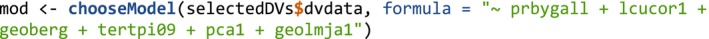

Model parameters are stored as “alpha” and “beta” following the notation of Elith et al. (2011) and Fithian and Hastie (2013). A vector of model predictions in probability ratio output (PRO) format is given by:

(1) $$\dot{q}=N\cdot e^{\alpha +\sum_{k=1}^{m}\beta_{k}x_{\mathrm{ik}}}$$

where *N* is the number of background locations, *α* is a normalizing constant, *β* is a vector of coefficients, and *x* is a matrix of DVs.

**Figure 4:**
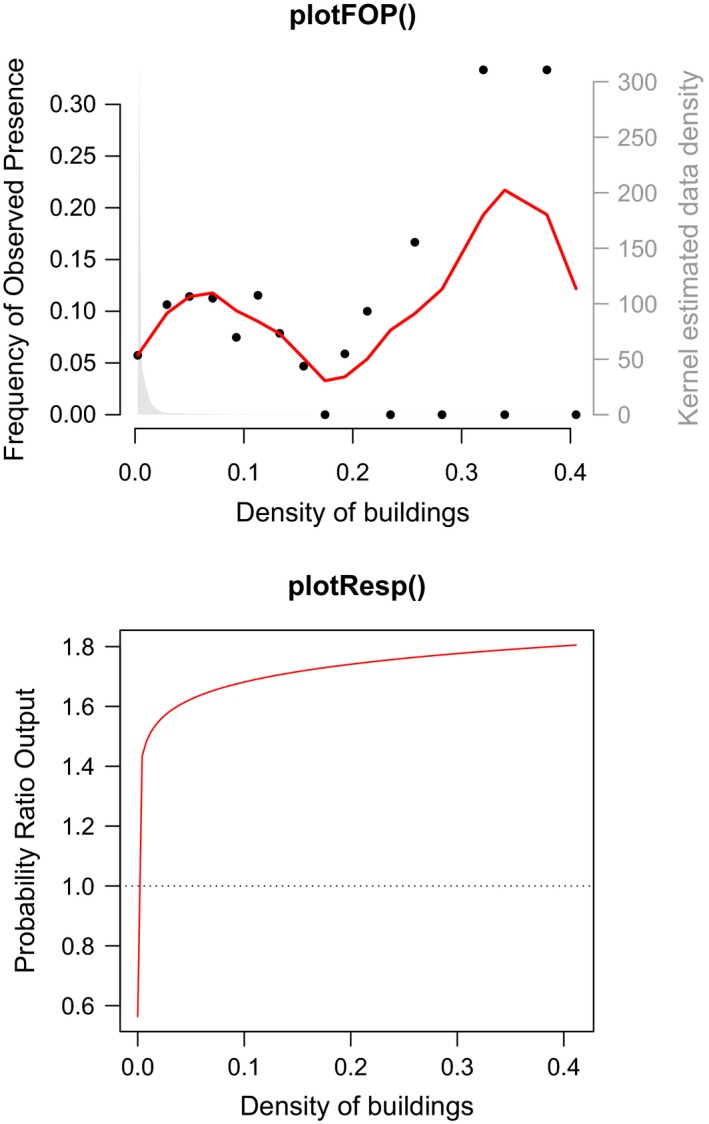
The frequency of observed presence plot (top) and single‐effect response plot (bottom) for the most important explanatory variable (EV) in the exemplified model

To assess how well the model captured empirical occurrence–environment relationships, response curves may be compared with their corresponding FOP plots (Figure 4). Note that, where the response curve deviates strongly from the pattern in FOP, data density is very low.

The projectModel() function produces model predictions from a model object, the transformations used to create its DVs, and any values of EVs:

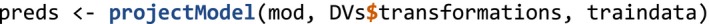

It also compares univariate ranges of the EV data in the projection to the ranges of the same EVs in the training data (scaled to [0,1]). Values of categorical EVs are classified as “inside” or “outside” the range of values present in the training data. Since we projected our model across the same data used to train it, all ranges are identical:

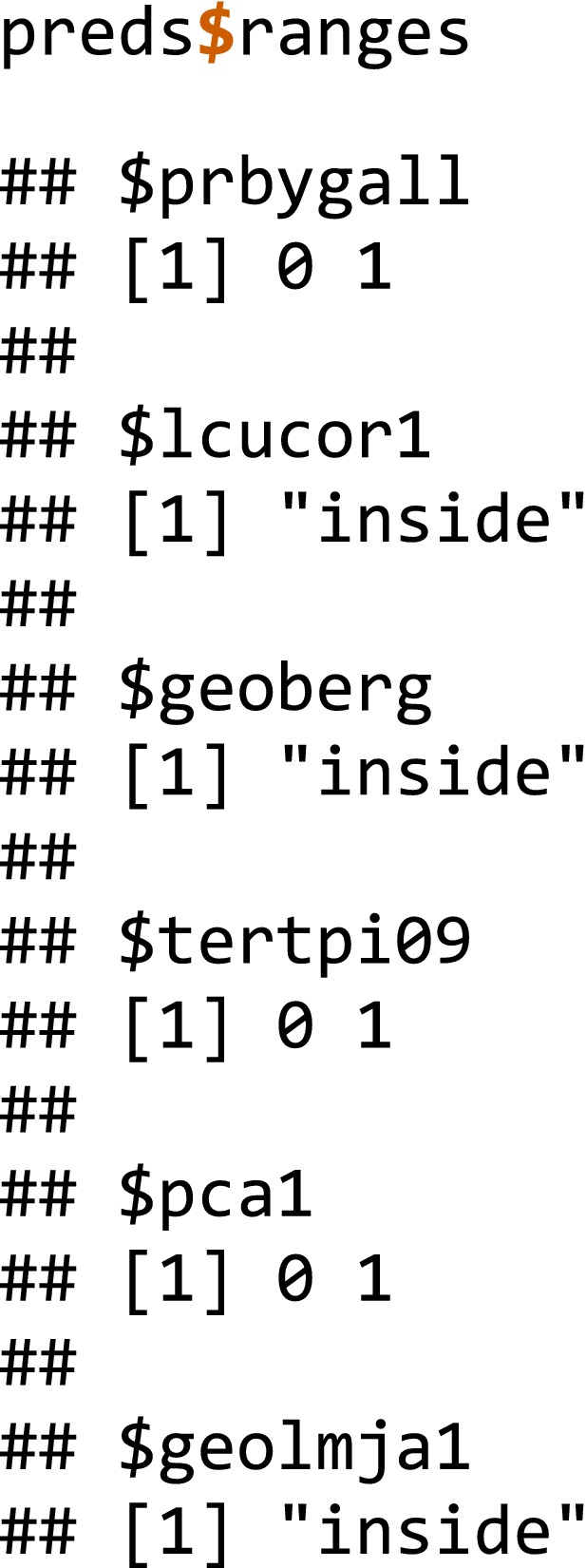

The testAUC() function calculates AUC for a given model based on evaluation data comprising occurrence data and the corresponding EV values. Preferably, the occurrence data include absences as well as presences. Plotting the receiver operating characteristic (ROC) curve is optional:

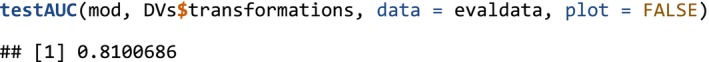

The workflow above builds maximum entropy models based on presence‐only occurrence data, through infinitely weighted logistic regression (IWLR). One useful feature of the MIAmaxent package is that the entire workflow can be adapted to presence–absence data and standard logistic regression (LR) models by changing a single setting (algorithm = “LR”) in functions that perform model fitting. Replacing three commands above with their counterparts below would result in a statistical approach identical in variable transformation and model selection, but with maximum entropy models replaced by logistic regression models:

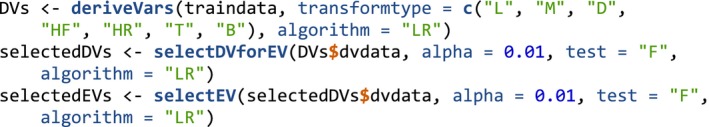

### 3.2 MIAmaxent‐maxnet comparison

In this section, we use an example data set to compare a maximum entropy model built using MIAmaxent (version 1.1.0) to one built using Maxent's R package equivalent, maxnet (version 0.1.2). As detailed above, the form of these models is identical (Appendix A3). The primary difference between the two approaches is in model selection, where MIAmaxent uses subset selection, while maxnet uses lasso regularization. Another smaller difference is that MIAmaxent automatically adds all presence locations to the background prior to maximum entropy model fitting by IWLR, because the background should be representative of all conditions in the study area (Halvorsen, 2012; Renner et al., 2015); maxnet does not add presences to the background. For increased comparability, we do not use threshold transformations or interaction terms in either MIAmaxent or maxnet.

We compare the resulting models in terms of two crucial, linked qualities: their discrimination ability and their complexity. It is useful to examine these two qualities together because there exists an optimal level of complexity that maximizes discrimination ability on holdout or independent data (Hastie et al., 2009). We quantify discrimination ability as AUC from spatially stratified cross‐validation. Spatially stratified cross‐validation is less likely than randomly partitioned cross‐validation to overestimate predictive performance due to spatial autocorrelation (Radosavljevic & Anderson, 2014; Veloz, 2009; Wenger & Olden, 2012), so it is a good way to assess predictive power when independent evaluation data are not available. We assess model complexity by the number of parameters and shapes of response curves (like Merow et al., 2014).

The example data we use are described in the paper that introduced Maxent (Phillips et al., 2006) and are available for download from https://biodiversityinformatics.amnh.org/open_source/maxent/. They consist of occurrence records for the brown‐throated three‐toed sloth (*Bradypus variegatus*), and 14 EVs representing climate, elevation, and potential vegetation categories. For both modeling approaches, we find an optimal level of model complexity by calculating AUC under various strengths of complexity penalty, as recommended by Merow et al. (2013). For the MIAmaxent approach, this means varying the significance threshold (alpha) in forward stepwise selection, and for the maxnet approach, it means varying the regularization multiplier.

**Figure 5:**
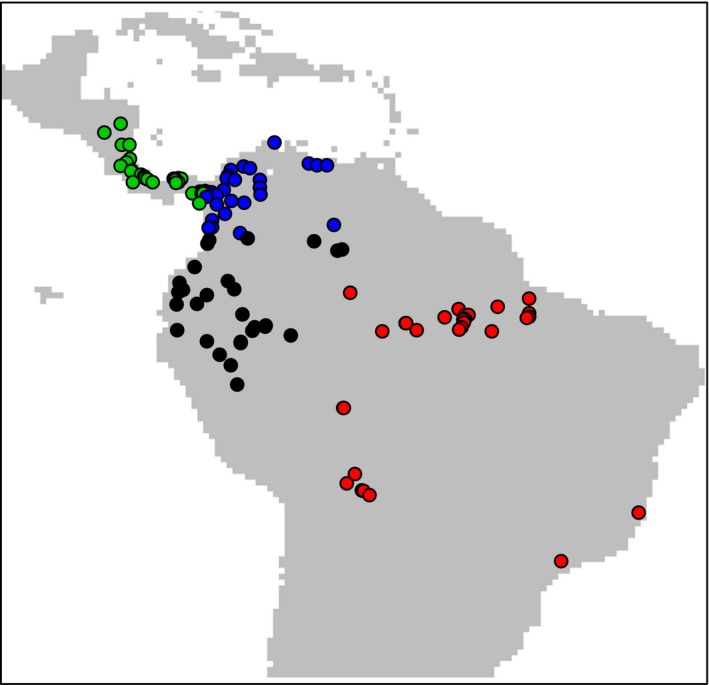
Presence locations of *Bradypus variegatus* in Central and South America, colored according their cross‐validation data partition, plotted across the modeling area

For spatially stratified cross‐validation, we group 114 presence records of *B. variegatus* into four data partitions (Figure 5). Specifically, we use the “block” partitioning method in the “ENMeval” package (version 0.3.0), which finds lines of latitude and longitude that divide the area into partitions holding equal numbers of records (Muscarella et al., 2014). Although not shown in the figure, uninformed background locations are partitioned into the same four geographic strata as the presences.

**Figure 6:**
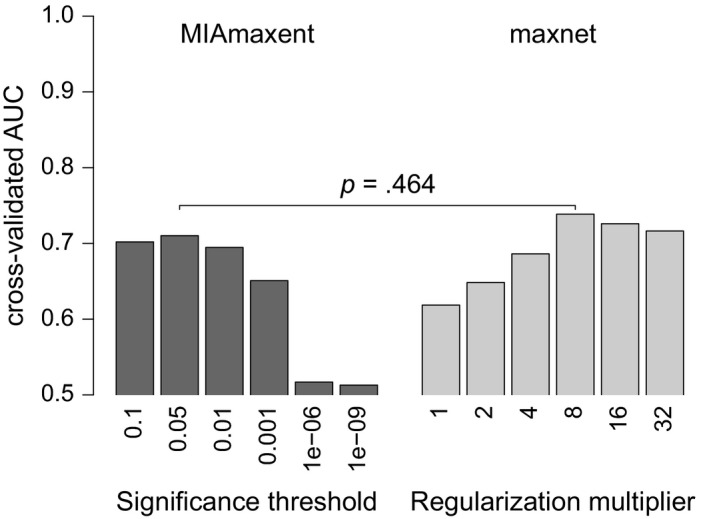
Discrimination abilities of distribution models of *Bradypus variegatus* produced by MIAmaxent (dark gray) and maxnet (light gray) under various strengths of complexity penalty calculated by spatially stratified cross‐validation. For the MIAmaxent and maxnet models of optimal complexity, the evidence against the hypothesis that their cross‐validated AUC values have equal population means (*p*) is weak

**Figure 7:**
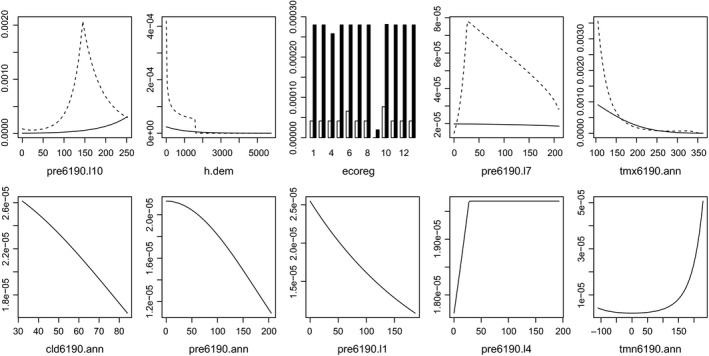
Marginal‐effect responses in distribution models of *Bradypus variegatus* made with MIAmaxent (dashed lines/empty bars) or maxnet (continuous lines/filled bars). The MIAmaxent model does not contain any of the explanatory variables in the bottom row. Responses are shown in “exponential” output (i.e., $\dot{q}/N$ in Equation 1). Scales of *y*‐axes differ across plots

Optimal model complexity is reached under a significance threshold of 0.05 in MIAmaxent and under a regularization multiplier of 8 in maxnet (Figure 6). The optimal MIAmaxent model shows marginally poorer AUC than the optimal maxnet model (mean difference: 0.028, 95% confidence interval for true difference in means from paired *t* test: [−0.080, 0.137]). The optimal MIAmaxent model contains 11 DVs representing 5 EVs, while the maxnet model contains 12 DVs representing 10 EVs (Figure 7; Appendix A4). In summary, the model produced by maxnet predicts marginally better than the model produced by maxnet, but it is less interpretable. However, spatially stratified cross‐validation does not completely eliminate spatial autocorrelation and shared sampling bias between training and test partitions, so measures of predictive performance obtained by this procedure may favor overfitted models (Merow et al., 2014). Therefore, independent test data might have shown the simpler MIAmaxent model to perform relatively better.

These results are in line with previous findings that models selected by subset selection maintain high‐predictive performance but are simpler and more ecologically meaningful than models selected by lasso regularization (e.g., Halvorsen et al., 2015, 2016). To illustrate, consider the response curves in Figure 7; the MIAmaxent model predicts highest occurrence of brown‐throated three‐toed sloth at an intermediate level of precipitation that is lower in July (pre6190.l10) than October (pre6190.l7), while the maxnet model predicts highest occurrence at precipitation levels that are low in January (pre6190.l1), high in April (pre6190.l4), low in July (pre6190.l7), high in October (pre6190.l10), and annually low (pre6190.ann). The response of the model produced by subset selection is clearly easier to justify ecologically.

## 4 INSTALLATION AND CITATION

The latest release version of the MIAmaxent package (currently version 1.1.0) can be installed from CRAN (Comprehensive R Archive Network) using the following command:

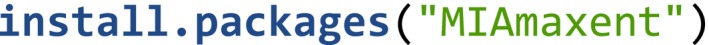

The package's GitHub page (https://github.com/julienvollering/MIAmaxent) may be used to report issues, contribute to the source code, or download the development version. To cite MIAmaxent, please cite this paper.

## 5 CONCLUSION

To summarize: of the three core modeling elements in the Maxent software, MIAmaxent (a) retains and slightly expands variable transformation, (b) retains maximum entropy fitting, with the option for standard logistic regression fitting, and (c) replaces lasso regularization with model selection by subset selection. MIAmaxent's decoupling of these three elements gives greater flexibility in statistical approaches and promotes fair methodological comparisons. For example, by providing a feasible alternative to Maxent's lasso regularization, MIAmaxent makes it easier to investigate whether this form of model selection is responsible for high‐predictive performance of models produced by Maxent. Similarly, MIAmaxent facilitates fair comparison between maximum entropy models built using presence‐only data and logistic regression models built using presence–absence data, by providing identical functionality for both. Modelers with access to both kinds of data sets may test which models show better performance, without confounding effects from differences in variable transformation or model selection (Guillera‐Arroita, Lahoz‐Monfort, & Elith, 2014). In other words, MIAmaxent can help disentangle the effect of the fitting algorithm from effects of other elements in the statistical approach. Asking why and when particular methodologies are advantageous is critical to advancing good distribution modeling practice (Elith & Graham, 2009).

Many authors have called for distributions modelers to consider more explicitly the ecological theory upon which their model is based (Austin, 2002, 2007; Elith & Leathwick, 2009; Halvorsen, 2012; Warren, 2012), and MIAmaxent grounds the practice of maximum entropy distribution modeling more strongly in ecological knowledge. MIAmaxent moves maximum entropy distribution models towards the “data modeling” school of statistical modeling (away from “algorithmic modeling”), by placing a higher premium on interpretable models (Breiman, 2001; Warren & Seifert, 2011). The most important change that MIAmaxent implements to cause this shift is to select models by subset selection instead of lasso regularization. Any distribution modeling approach—including how the model is produced and how it is evaluated—must be adapted to the purpose of the study and the characteristics of the data (Halvorsen, 2012; Merow et al., 2014), as no single approach is most suitable for all studies (Escobar, Qiao, Cabello, & Peterson, 2018; Mazzoni, 2016; Qiao, Soberón, & Peterson, 2015). MIAmaxent expands a distribution modeler's statistical toolbox, and for studies aiming to do something other than predict with minimal error the geographic distribution of the modeled target in the same spatial and temporal context as the data, MIAmaxent may frequently be more suitable than the Maxent software.

## CONFLICT OF INTERESTS

The authors declare no conflicts of interest.

## AUTHOR CONTRIBUTIONS

JV designed and coded the MIAmaxent package and drafted the paper. RH conceived the theoretical approach underlying the package and critically revised the paper. SM conceived the MIAmaxent package and developed its underlying informatics approach, provided data for the example, and reviewed the paper.

## Figures and Tables

**Figure A1:**
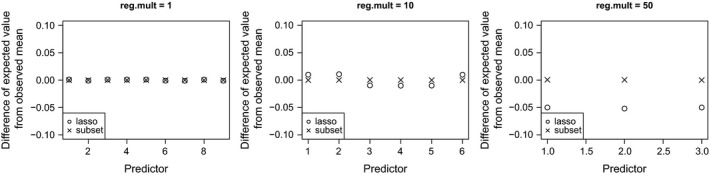
Deviation of expected values of predictions from the observed mean in the data, under three regularization scenarios (maxnet lasso regularization multiplier = 1, 10, 50). Deviations in models using lasso regularization are compared with deviations in unregularized models using the same set of predictors

**Figure A2:**
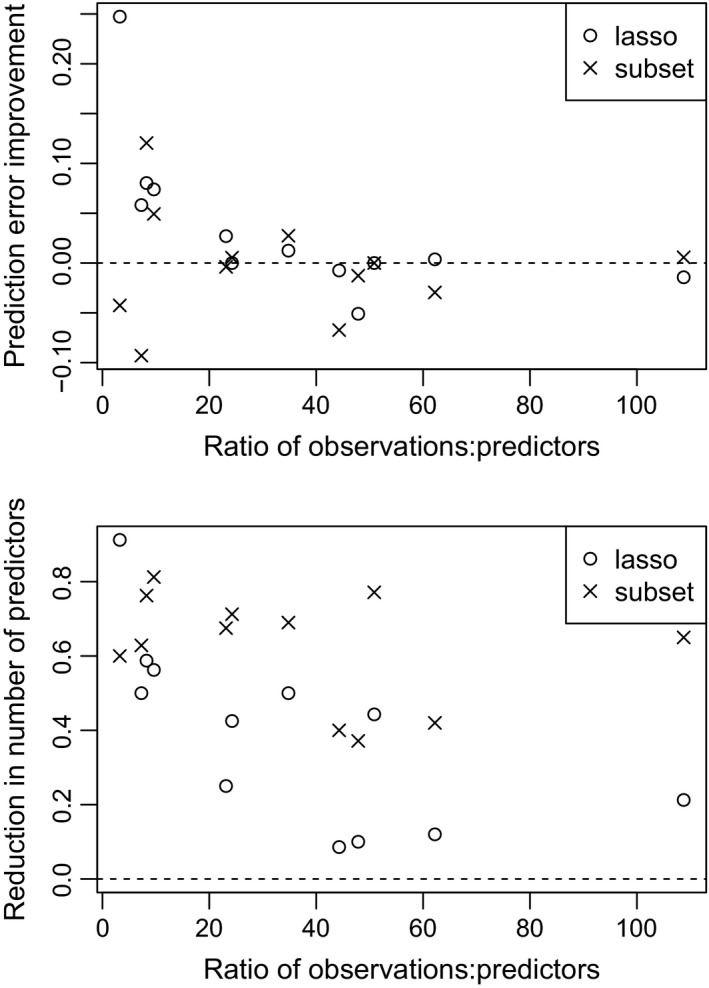
Improvement in mean squared prediction error (top panel) and reduction in number of predictors (bottom panel) by lasso regularization and subset selection, as the ratio of observations to candidate predictors increases. Note that, higher values on the y‐axis in the bottom panel represent simpler models. Data from table 1 in Dahlgren, 2010

**Figure A3:**
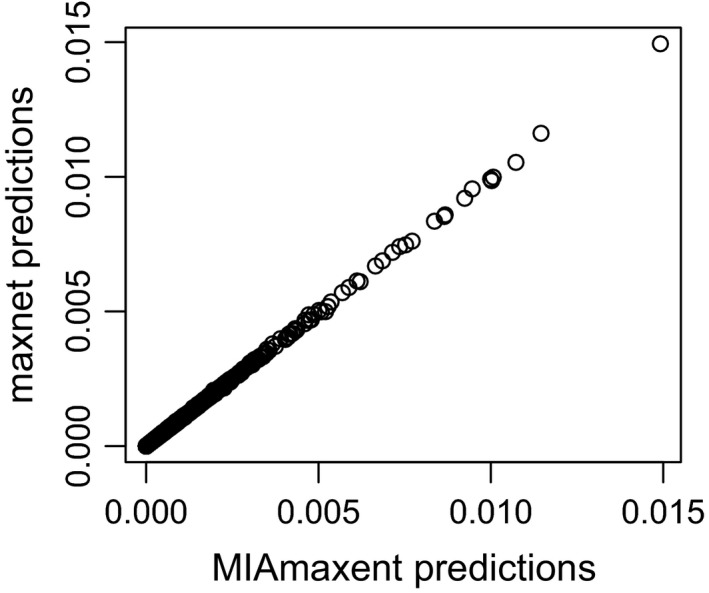
Near equivalence of “MIAmaxent” predictions and “maxnet” predictions, when maxnet uses a regularization multiplier close to zero and presence observations as part of the background. This illustration uses the “bradypus” data set from the maxnet package

## Data Availability

The data used in the “MIAmaxent workflow” example accompany the MIAmaxent package (version 1.1.0), while those used in the “MIAmaxent‐maxnet comparison” accompany the maxnet package (version 0.1.2). Both are available from the Comprehensive R Archive Network (https://cran.r-project.org/). This whole paper is fully reproducible from an R‐markdown file that is available on GitHub (https://github.com/julienvollering/MIAmaxent-paper).

## APPENDIX A

### A1: Deviation of expected values from empirical values under increasing lasso regularization

Regularization loosens the constraint on the maximum entropy distribution that the expected value of each predictor must match its observed mean. As a result, increasing the regularization multiplier to decrease model complexity has the effect that predictions are no longer fully consistent with the data (Merow et al., 2013, see also accompanying figure D2 in appendix 4). Subset selection does not have the same effect (Figure A1).

### A2: Model building using subset or shrinkage methods

Dahlgren (2010) presented shrinkage methods and particularly the lasso as good alternatives to subset selection methods for model building with ecological data when the data comprise few observations and many candidate explanatory variables. He also advocates for avoiding model building altogether by using full models when the number of observations is sufficiently high and there is no strong collinearity between variables. Our plotting of his Table 1 results (Figure A2) suggests that, in data sets of more than 20–30 observations per candidate explanatory variable, neither the lasso nor subset selection strongly improves prediction error compared with the full model, but subset selection results in more interpretable and parsimonious models than the lasso.

### A3: Conditional equivalence of maximum entropy models in “MIAmaxent” and “maxnet” packages

If presence observations are included in the background manually, and regularization is close to zero, predictions from models built using the “maxnet” package are near equivalent to predictions from models built using the “MIAmaxent” package (Figure A3). Note that, regularization cannot be turned off entirely in the maxnet package.

### A4: Parameters of optimal models in “MIAmaxent‐maxnet comparison” section

See Tables A1 and A2.

**Table A1:** Parameters of the optimal MIAmaxent model in the “MIAmaxent‐maxnet comparison” section

	Value
pre6190.l10_HR12	−9.5090627
pre6190.l10_M	−8.3016253
h.dem_M	−2.5362354
h.dem_HF6	−5996.8673896
ecoreg_BX10	0.6126549
ecoreg_BX6	0.4597000
ecoreg_BX9	−19.8281359
pre6190.l7_HR3	−1.7611179
pre6190.l7_D05	1.1769038
tmx6190.ann_D2	3.8821114
tmx6190.ann_HF18	−2.8850423
alpha	0.1228392

**Table A2:** Parameters of the optimal maxnet model in the “MIAmaxent‐maxnet comparison” section

	Value
h.dem	−0.0009343
pre6190.l1	−0.0045560
pre6190.l10	0.0161984
I(cld6190.ann^2^)	−0.0000770
I(pre6190.ann^2^)	−0.0000158
I(pre6190.l7^2^)	−0.0000015
I(tmn6190.ann^2^)	0.0000607
I(tmx6190.ann^2^)	−0.0000440
hinge(pre6190.l4):0:27.5714285714286	0.1078618
categorical(ecoreg):4	−0.0809360
categorical(ecoreg):9	−2.6541785
categorical(ecoreg):10	0.0045407
alpha	−6.6204540
